# Effect of preoperative sleep quality on rapid postoperative recovery from non-traumatic rotator cuff injuries

**DOI:** 10.3389/fmed.2025.1587685

**Published:** 2025-10-10

**Authors:** Limin Mou, Wenhao Zhang, Jun Liao, Siping Zhang, Lingling Zhao, Remila Aimaiti, Munire Abuduxiku, Wenyuan Xiang, Yingjie Deng, Rui Fang

**Affiliations:** The Fourth Clinical Medical College of Xinjiang Medical University (Hospital of Traditional Chinese Medicine Affiliated to Xinjiang Medical University), Ürümqi, Xinjiang, China

**Keywords:** sleep quality, rotator cuff tear, shoulder arthroscopy, rapid rehabilitation, pain, anxiety, depression

## Abstract

**Background:**

Global aging has led to a continuous increase in rotator cuff injuries, often accompanied by clinical-related sleep disorders. There is a lack of clinical research on whether preoperative sleep quality affects rapid postoperative recovery; therefore, it is necessary to conduct clinical follow-up studies to explore the relationship between preoperative sleep quality and postoperative rehabilitation, in order to provide rehabilitation guidance for clinical practice.

**Methods:**

In this prospective cohort study, 256 patients who underwent arthroscopic rotator cuff repair (ARCR) at our center from January 2022 to January 2024 were grouped based on the Pittsburgh Sleep Quality Index (PSQI): the high sleep quality group (HSQG, PSQI < 7, *n* = 120) and low sleep quality group (LSQG, PSQI ≥ 7, *n* = 136). All patients were given the same rapid rehabilitation protocol. Before the operation and on the 1st, 3rd, 1st week, 1st month, 3rd month, and 6th month after the operation, visual analog scale (VAS) pain scores, Self-Rating Anxiety Scale (SAS) score, Self-Rating Depression Scale (SDS) score, American Shoulder and Elbow Surgeons (ASES) score, Constant-Murley score, and patient satisfaction were evaluated. At the same time, the occurrence of adhesions, re-tears, and other complications was recorded.

**Results:**

All 256 cases successfully completed arthroscopic repair and received complete follow-up. There were no statistically significant differences in baseline data, pain and function scores between the two groups (*p* > 0.05). Early stage: In the first day, third day and first week after surgery, the VAS, SAS and SDS scores of the HSQG were significantly lower than those of the LSQG (*p* < 0.05). Mid-term: At 1, 3, and 6 months after surgery, the differences in the above indicators disappeared (*p* > 0.05). Function: The ASES and Constant-Murley scores of the HSQG were better than those of the LSQG from 1 week to 3 months after surgery (*p* < 0.05), and there was no difference between the two groups at other time points. Satisfaction: The hospital satisfaction of the HSQG was significantly higher (*p* < 0.05). Complications: The incidence of LSQG was 5.9%, and that of high sleep quality group was 3.3%. There was no statistically significant difference (*p* > 0.05).

**Conclusion:**

Maintaining good sleep quality before surgery can alleviate early postoperative pain, reduce the risk of postoperative anxiety and depression, accelerate functional recovery, and enhance patient satisfaction. It is recommended to incorporate preoperative sleep optimization into the rapid rehabilitation process for rotator cuff injuries to facilitate patients’ rapid postoperative recovery.

## Introduction

Rotator cuff tear (RCT) is the leading cause of shoulder pain and dysfunction in adults, with its incidence rate continuously increasing along with the global aging process (1–4). Degenerative RCT often progresses from partial tears of a single layer to full-thickness defects, accompanied by tendon retraction and persistent pain, eventually requiring surgical reconstruction to restore anatomy and function (5, 6). Arthroscopic rotator cuff repair (ARCR), due to its minimally invasive nature and rapid recovery, has become the gold standard for patients who fail conservative treatment (7, 8).

Sleep disorders are common comorbidities in RCT patients. Nighttime pain significantly disrupts sleep, and insufficient sleep exacerbates pain perception through central sensitization and hinders postoperative functional recovery by reducing rehabilitation compliance (9–11). This “pain-insomnia-negative emotion” vicious cycle not only affects the quality of life of patients but may also weaken the efficacy of ARCR (12–14). However, current research mostly focuses on postoperative sleep conditions, and there is a lack of systematic evidence regarding the role of preoperative sleep quality in rapid recovery, especially in the elderly population. Therefore, this study aims to evaluate the predictive value of preoperative sleep quality on early postoperative pain, functional recovery, and psychological status in middle-aged and elderly RCT patients, with the goal of providing evidence-based guidance for perioperative individualized management.

## Materials and methods

### General information

The research subjects were selected from consecutive cases that underwent arthroscopic supraspinatus tendon repair in our hospital from January 2022 to January 2024. All patients received a uniform intravenous general anesthesia and postoperative patient-controlled analgesia pump protocol.

### Inclusion criteria

1. MRI confirmed rotator cuff injury was limited to the supraspinatus tendon, and Patte classification was grade I-III.
2. Preoperative passive range of motion of the shoulder joint was not restricted.
3. Age 40–65 years old, and mental health (no depression or anxiety).
4. No use of benzodiazepines, sedatives or antidepressants and other mental/sleep-related drugs within 1 week before surgery.
5. Complete clinical and follow-up data, with a follow-up period of ≥ 6 months.
6. Voluntary signing of informed consent.

### Exclusion criteria

1. Comorbidities such as diabetes, hypertension, coronary heart disease or hyperlipidemia.
2. Preoperative shoulder joint stiffness, labral or other rotator cuff tendon injuries.
3. Calcific tendinitis or other immune diseases.
4. History of trauma, surgery or mental illness in the affected limb.
5. Incomplete preoperative data or refusal to follow up.

After independent review by two senior attending physicians, a total of 256 patients met all the above conditions. All patients were diagnosed and evaluated by sleep experts and classified into a HSQG (PSQI < 7 points, *n* = 120) and a LSQG (PSQI ≥ 7 points, *n* = 136) based on the PSQI score. The baseline characteristics of the two groups were balanced and comparable: the HSQG had 45 males and 75 females, with an average age of 54.93 ± 5.65 years and an average disease duration of 9.39 ± 3.01 months; the LSQG had 61 males and 75 females, with an average age of 53.91 ± 6.06 years and an average disease duration of 9.06 ± 3.61 months.

### Preoperative preparation

On the day of admission, two senior shoulder joint surgeons conducted a systematic assessment of the patient. First, a complete medical history was collected, followed by a physical examination and a review of preoperative images to confirm the surgical indications and eligibility for inclusion in the study. After the assessment, the research team provided the patient with detailed instructions on perioperative precautions and conducted standardized psychological counseling to alleviate preoperative anxiety and enhance treatment confidence. All baseline data were recorded before the operation and archived in groups.

### Surgical method and postoperative management

After successful anesthesia, the patient was placed in a beach chair position. The surgical field was routinely disinfected and draped, and the bony landmarks such as the clavicle, acromial tuberosity, and coracoid process were marked with methylene blue. The posterolateral approach was located approximately 2 cm below the posterior lateral angle of the acromion, and an 8.5 mm joint camera cannula was inserted. The glenohumeral joint was systematically examined: the integrity of the labrum, the subscapularis tendon, the long head of the biceps tendon, the coracoid process tendon, and the state of the joint cartilage were evaluated. Then, a standard anterior approach was established, using a shaver to remove the hyperplastic synovium and radiofrequency to stop the bleeding. The anterior lateral approach was entered into the subacromial space. The shoulder-shin splinting sign was dynamically observed. If necessary, acromioplasty was performed. After completely clearing the synovium around the supraspinatus muscle, the probe was used to assess the size, shape, and retraction degree of the tear, and to confirm that the fracture ends could cover the footprint area without tension. The footprint area was freshened with a burr. The repair was performed using the double-row suture-bridge technique: The inner row: 2 full suture anchors (Ti-alloy, Beijing Tianxing Medical, China Medical Device Registration No. 20193130262) were implanted at the cartilage margin, and the suture was passed through the tendon using the modified Mason-Allen method; The outer row: 2 absorbable bone anchors (PLLA) were placed laterally at the distal end of the greater tuberosity, and the tail line of the inner row was tied and fixed with a shaver to achieve full coverage of the footprint area without gaps. Under the microscope, after confirming that the shoulder cuff tension was moderate and the fixation was reliable, the joint cavity was rinsed and thoroughly stopped bleeding. The incision was closed layer by layer. 10 ml of 0.2% ropivacaine and 5 mg of compound betamethasone were injected into the joint cavity. After the operation, the shoulder joint was fixed with a 30° abduction brace.

All surgeries were performed by the same senior chief physician following a unified protocol. During the perioperative period, the accelerated recovery (ERAS) pathway was followed:

- Prevention of infection: a single intravenous antibiotic within 24 h after surgery.
- Multimodal analgesia: after 48 h of patient-controlled analgesia pump usage, the medication was changed to oral ketoprofen and mefenamic acid 12.5 mg every 12 h after meals.
- Local treatment: intermittent ice application (20 min per time, 4–6 times per day), and specific electromagnetic spectrum (TDP) and carbon photon therapy were administered around the incision starting from the first day after surgery (20 min, 1–2 times per day).
- Early rehabilitation (Day 1): active flexion and extension of the hand and forearm; passive activities such as Codman pendulum and circle movements were performed in the supine position under the guidance of a rehabilitation therapist, with the shoulder joint maintained at 15–30° of abduction and external rotation, and 90° elbow flexion with a brace fixation, and weight-bearing was prohibited.
- Advanced training (6–12 weeks): gradually transitioning to active-assisted and active joint movements (climbing walls, shrugging shoulders, internal and external rotation), restoring full range of motion in the scapular plane and reconstructing passive tension.
- Discharge education: standardized rehabilitation videos were provided before discharge, clearly outlining the 6-month staged rehabilitation plan and precautions.
- Follow-up: outpatient rechecks were conducted at 1 week, 1 month, 3 months, and 6 months after surgery, with ultrasound assessment of rotator cuff healing, quantification of function using ASES and Constant-Murley scores, and all data were recorded for follow-up.

### Observation indicators

Prospective records were made of the general patient information, operation time, number of anchors, hospital stay, size of rotator cuff tear, and hospital satisfaction. Before the operation and at each follow-up, the ASES score, Constant-Murley score, and VAS for pain were used to assess the function, and the SAS and SDS were used to evaluate the psychological state; postoperative complications were continuously recorded throughout the follow-up period.

All the scales were completed by two shoulder joint specialists who had undergone standardized training under a blinded condition. Hospital satisfaction: A 5-point Likert scale was used, with 1 point indicating “extremely dissatisfied” and 5 points indicating “very satisfied.” The higher the score, the higher the satisfaction (15). ASES score: The total score is 100 points, with pain and daily life each accounting for 50%. The higher the score, the better the shoulder joint function (16). Constant-Murley score: The total score is 100 points, including 15 points for pain, 20 points for daily life, 25 points for strength, and 40 points for range of motion; a score >90 is excellent, 80–89 is good, 70–79 is fair, and <70 is poor (17). VAS pain: A 10-cm ruler method, 0–100 mm, with lower scores indicating less pain (18). SAS and SDS: Each contains 20 items, SAS includes 5 positive and 15 negative items, and SDS is all negative items. <50 points indicate normal, 50–59 points indicate mild, 60–69 points indicate moderate, and ≥70 points indicate severe anxiety/depression (19, 20). All scales were filled out by the patients after adequate information was provided, and the answer sheets were cross-checked and agreed upon by two experts; ASES and Constant-Murley scores were averaged by the two assessors.

### Statistical methods

Statistical analysis was performed with IBM SPSS 26.0 and visualized using GraphPad Prism 9.5 and Excel. Continuous variables exhibiting normal distribution are presented as mean ± SD. Categorical data are expressed as percentages and compared using the χ^2^ test or Fisher’s exact test as appropriate. Between-group differences in continuous variables were examined with independent-sample t tests, and effect size was quantified using Cohen’s d (|d| ≥ 0.80 considered large). Repeated-measures ANOVA was employed for longitudinal comparisons across multiple postoperative time points; sphericity was assessed with Mauchly’s test, and the Greenhouse–Geisser correction was applied when necessary. Partial eta squared (ηp^2^) was calculated as the effect size for within-subject factors (ηp^2^ ≥ 0.14 interpreted as large). *Post-hoc* pairwise comparisons were conducted with Bonferroni correction. Within-group pre- versus post-operative changes were analyzed by one-way ANOVA. A two-tailed *p-*value < 0.05 was considered statistically significant. All statistical procedures were reviewed and approved by the Department of Biostatistics, The Fourth Clinical Medical College of Xinjiang Medical University.

## Results

**Figure 1:**
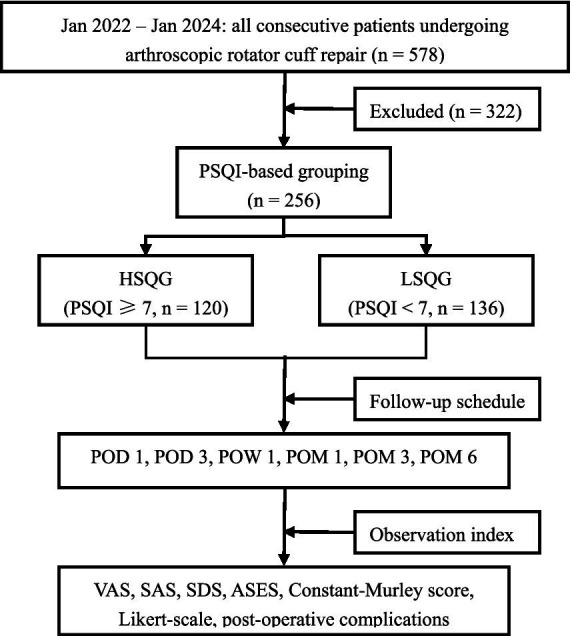
Patient screening and study follow-up flow chart. PSQI, Pittsburgh Sleep Quality Index; HSQG, High sleep quality group; LSQG, Low sleep quality group; POD, postoperative day; POW, postoperative week; POM, postoperative month; VAS, Visual Analog Scale; SAS, Self-Rating Anxiety Scale; ASES, American Shoulder and Elbow Surgeons.

As shown in Figure 1, from January 2022 to January 2024, 578 patients scheduled for arthroscopic rotator cuff repair (ARCR) were continuously screened. Eventually, 256 patients met the inclusion criteria and completed the follow-up. Three hundred and twenty-two patients were excluded, mainly due to: shoulder joint stiffness (*n* = 62), combined labrum or other tendon injuries (*n* = 52), trauma history (*n* = 61), recent 1-week use of psychotropic or sleep medications (*n* = 57), underlying diseases such as hypertension or diabetes (*n* = 51), and refusal or loss to follow-up (*n* = 39). The 256 patients were divided into a high sleep quality group (PSQI ≥ 7, *n* = 120) and a low sleep quality group (PSQI < 7, *n* = 136) based on the Pittsburgh Sleep Quality Index (PSQI). Both groups were managed by the same surgical team with the same perioperative management, and their pain (VAS), emotions (SAS/SDS), function (ASES and Constant-Murley), hospital satisfaction (Likert), and complications were evaluated before surgery, on POD 1, POD 3, POW 1, POM 1, POM 3, and POM 6. Although the exclusion of patients with underlying diseases may limit extrapolation, the strict homogeneity design enhances internal validity.

### Comparison of general data

There were no statistically significant differences between the two groups of patients in terms of preoperative age, disease duration, BMI, gender, affected side, and Patte classification (all *p* > 0.05). There were also no significant differences in postoperative surgical time, rivets, and length of hospital stay (all *p* > 0.05). These results indicate that the two groups are highly comparable (Table 1).

**Table 1:** Comparison of baseline data in each group.

Group	Age	Dis Dur	LOS	BMI	Surg time	Rivets	Gender (M/F)	Side (L/R)	Patte (I/II/III)
High sleep quality group (*n* = 120)	54.93 ± 5.65	9.39 ± 3.01	4.73 ± 0.96	25.93 ± 1.06	54.24 ± 7.67	3.58 ± 0.49	45/75	42/78	22/92/6
Low sleep quality group (*n* = 136)	53.91 ± 6.06	9.06 ± 3.61	4.69 ± 0.97	25.83 ± 1.17	53.54 ± 8.34	3.55 ± 0.48	61/75	55/81	20/110/6
T/χ^2^	1.38	0.79	0.28	0.68	0.69	0.51	1.42	0.80	5.81
*p-*value	0.17	0.43	0.77	0.49	0.48	0.61	0.23	0.37	0.06

Age (years); Dis Dur, Disease duration (months); LOS, length of hospital stay (days); BMI, body-mass index (kg/m^2^); Surg Time, surgical time (min); Rivets, number of suture anchors used; Gender (M, male; F, female); Side (L, left; R, right); Patte, tear classification (I/II/III).

### Comparison of VAS scores

**Figure 2:**
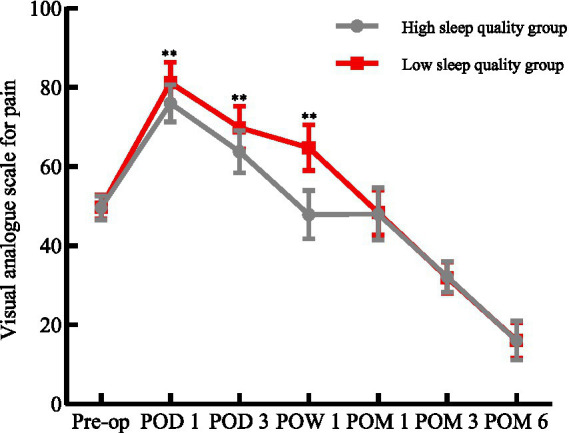
Comparison of VAS Scores between the two groups. Pre-op, preoperative; POD, postoperative day; POW, postoperative week; POM, postoperative month; VAS, visual analogue scale (0–100 mm); ^**^p < 0.01 for comparisons between the HSQG and LSQG at the same time point. Data are shown as mean ± SD.

There was no difference in VAS scores between the two groups before the operation (*p* > 0.05). On the 1st day, 3rd day, and 1st week after the operation, the pain level of HSQG was significantly lower than that of LSQG (*p* < 0.01); after that, the difference disappeared (*p* > 0.05). The pain levels of both groups reached their peak on the 1st day after the operation and then continued to decline. At 6 months, they were significantly better than the baseline (Figure 2). Repeated measures analysis of variance confirmed a significant time main effect (Wilks’ *Λ* = 0.008, *F*₆,₂₄₉ = 5035.89, *p* < 0.05, η^2^p = 0.992) and a significant time × group interaction effect (Wilks’ Λ = 0.300, *F*₆,₂₄₉ = 96.72, *p* < 0.05, η^2^p = 0.700), indicating that preoperative sleep quality can affect postoperative pain.

### Comparison of SAS scores

**Figure 3:**
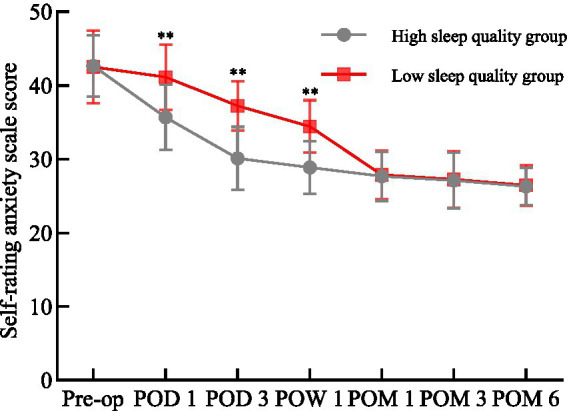
Comparison of SAS scores between the two groups. Pre-op, preoperative; POD, postoperative day; POW, postoperative week; POM, postoperative month; ^**^p < 0.01 for comparisons between the HSQG and LSQG at the same time point. Data are shown as mean ± SD.

There was no difference in the SAS scores between the two groups before the operation (*p* > 0.05). On the 1st day, 3rd day, and 1st week after the operation, the anxiety level of HSQG was significantly lower than that of LSQG (*p* < 0.01); after that, the difference disappeared (*p* > 0.05). The SAS scores of both groups showed a downward trend from before the operation to 6 months, and stabilized after 1 month. At 6 months, anxiety was significantly improved compared to the baseline (Figure 3). Repeated measures variance analysis confirmed a significant time main effect (Wilks’ *Λ* = 0.055, *F*₆,₂₄₉ = 715.52, *p* < 0.05, η^2^p = 0.945) and a significant time × group interaction effect (Wilks’ Λ = 0.517, *F*₆,₂₄₉ = 38.80, *p* < 0.05, η^2^p = 0.483), indicating that preoperative sleep quality can affect postoperative anxiety.

### Comparison of SDS scores

**Figure 4:**
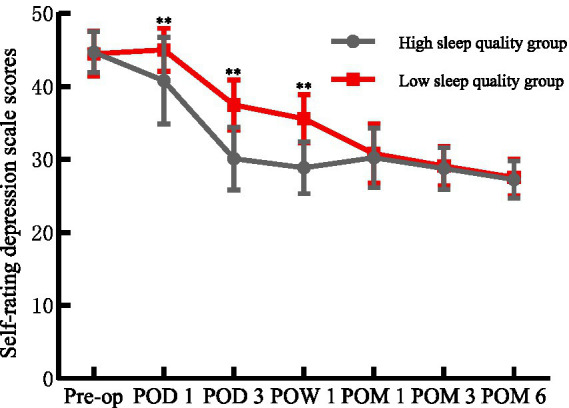
Comparison of SDS scores between the two groups. Pre-op, preoperative; POD, postoperative day; POW, postoperative week; POM, postoperative month; ^**^p < 0.01 for comparisons between the HSQG and LSQG at the same time point. Data are shown as mean ± SD.

There was no difference in the SDS scores between the two groups before the operation (*p* > 0.05). On the 1st day, 3rd day, and 1st week after the operation, the depression score of HSQG was significantly lower than that of LSQG (*p* < 0.05), and the difference disappeared later (*p* > 0.05). The SDS scores of both groups showed a downward trend from before the operation to 6 months, and at 6 months, they were significantly improved compared to the baseline (Figure 4). Repeated measures variance analysis showed that there was a significant time main effect (Wilks’ *Λ* = 0.055, *F*₆,₂₄₉ = 715.52, *p* < 0.05, η^2^p = 0.945) and a significant time × group interaction effect (Wilks’ Λ = 0.445, *F*₆,₂₄₉ = 51.70, *p* < 0.05, η^2^p = 0.555), indicating that the preoperative sleep quality could affect postoperative depression.

### Comparison of ASES scores

There was no significant difference in the ASES scores between the two groups before the operation (*p* > 0.05). On the first day after the operation and at 6 months, there was still no statistical difference in the scores between the two groups (*p* > 0.05). On the first day and 1 week after the operation, the scores of both groups were lower than the baseline levels, but they significantly improved at 1, 3, and 6 months. It is noteworthy that the ASES scores of the HSQG were better than those of the LSQG at 1 week, 1 month, and 3 months after the operation (*p* < 0.05), with effect sizes of small, medium, and small (Cohen’s *d* = 0.31, 0.42, 0.29). The details are shown in Table 2. This result suggests that high-quality sleep is conducive to promoting the early functional recovery after ARCR surgery.

**Table 2:** Comparison of ASES score results between groups.

Group	Pre-op	POD 1	POW 1	POM 1	POM 3	POM 6
High sleep quality group (*n* = 120)	42.65 ± 4.15	28.08 ± 4.50	32.07 ± 4.42	49.74 ± 3.62^*^	78.23 ± 4.78^*^	82.52 ± 1.82^*^
Low sleep quality group (*n* = 136)	42.51 ± 4.93	27.43 ± 3.86	30.71 ± 3.97	48.07 ± 4.29^*^	76.67 ± 5.86^*^	82.13 ± 1.37^*^
*T*	0.24	1.24	2.61	3.35	2.31	1.96
*P-*value	0.81	0.22	0.01	<0.05	0.02	0.05
Cohen’s *d* (95% Cl)	–	–	0.31 (0.05–0.57)	0.42 (0.16–0.68)	0.29 (0.03–0.55)	–

### Comparison of Constant-Murley scores

There was no significant difference in preoperative Constant-Murley scores between the two groups (*p* > 0.05). Similarly, no significant differences were observed at postoperative 1 day and 6 months (*p* > 0.05). At 1 day, 1 week, and 1 month after the operation, the scores of both groups were lower than those before the operation, but they significantly improved at 3 months and 6 months. It is noteworthy that the Constant-Murley score of the HSQG was significantly higher than that of the LSQG at 1 week, 1 month, and 3 months after the operation (*p* < 0.05), with effect sizes of small, medium, and medium (Cohen’s *d* = 0.28, 0.40, 0.40). The details are shown in Table 3. This indicates that good sleep quality helps promote postoperative functional recovery and accelerates the rehabilitation process.

**Table 3:** Comparison of Constant-Murley score results between groups.

Group	Pre-op	POD 1	POW 1	POM 1	POM 3	POM 6
High sleep quality group (*n* = 120)	52.64 ± 3.73	29.24 ± 5.06	33.30 ± 3.79	52.22 ± 3.36	78.83 ± 3.96^*^	82.85 ± 1.96^*^
Low sleep quality group (*n* = 136)	51.94 ± 4.73	28.44 ± 4.13	32.17 ± 4.39	50.75 ± 3.98	76.93 ± 5.23^*^	82.52 ± 1.87^*^
*T*	1.29	1.39	2.17	3.16	3.23	1.36
*P-*value	0.19	0.16	0.03	<0.05	<0.05	0.17
Cohen’s *d* (95% Cl)	–	–	0.28 (0.02–0.54)	0.40 (0.14–0.66)	0.40 (0.14, 0.66)	–

Pre-op, preoperative; POD, postoperative day; POW, postoperative week; POM, postoperative month; Constant-Murley Shoulder Score (0–100); Data are mean ± SD; ^*^P<0.05,compared with preoperative.

### Comparison of hospital satisfaction

**Figure 5:**
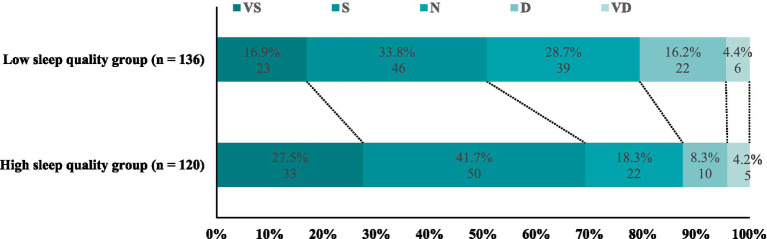
Comparison of hospital satisfaction between the two groups. Patient-reported global satisfaction at discharge. Categories: VS, very satisfied; S, satisfied; N, neutral; D, dissatisfied; VD, very dissatisfied.

As shown in Figure 5, there is a significant difference in the distribution of Likert-scale satisfaction between the two groups. In the HSQG, 27.5% of the patients expressed “very satisfied,” and 41.7% expressed “satisfied”; while in the LSQG, the corresponding proportions were only 16.9 and 33.8%. The proportion of “unsatisfied” in the HSQG was 8.3%, significantly lower than 16.2% in the LSQG. The difference in Likert scale distribution between the two groups was statistically significant (χ^2^ = 10.32, *p* = 0.03). The results indicate that the higher the preoperative sleep quality, the higher the satisfaction during hospitalization, which may promote rapid postoperative recovery.

### Comparison of postoperative complications

During the follow-up period, neither of the two groups of patients experienced infection, fracture or neurovascular injury. In the HSQG, 2 cases (1.7%) of rotator cuff re-tear occurred 1–3 months after the operation, and 2 cases (1.7%) of shoulder joint adhesion occurred 6 months after the operation; in the LSQG, 3 cases (2.2%) of rotator cuff re-tear occurred 3 months after the operation, and 5 cases (3.7%) of shoulder joint adhesion occurred 6 months after the operation. The total complication rate in the LSQG was slightly higher (5.9% vs. 3.3%), but there was no statistical significance (χ^2^ = 0.17, *p* = 0.68) (Table 4). All of the above complications were well controlled through reoperation or individualized rehabilitation treatment, and the function of the affected limb recovered to a near-normal level.

**Table 4:** Comparison of postoperative complications between the two groups of patients.

Group	Inf	NVI	Fx	RCR	JA	Inc(%)
High sleep quality group (*n* = 120)	0	0	0	3	5	5.9
Low sleep quality group (*n* = 136)	0	0	0	2	2	3.3
χ^2^	–	–	–	–	–	0.17
*P-*value	–	–	–	–	–	0.68

Inf, infection; NVI, neurovascular injury; Fx, fracture; RCR, rotator cuff re-tear; JA, joint adhesion; Inc, incidence rate (%).

## Discussion

With the aging population and the rise of national fitness campaigns, the number of patients with rotator cuff injuries has been increasing annually. Currently, RCT have become a major cause of upper limb dysfunction and sleep disturbances in patients (21). Sleep, as a fundamental physiological need, is considered a dynamic form of rest and is crucial for maintaining physical and mental health. Good sleep facilitates metabolic regulation in the body, and high-quality sleep not only benefits neuroimmune regulation but also reduces the body’s sensitivity to pain (22, 23). Research has shown that poor sleep quality can increase the risk of various chronic conditions, such as diabetes, depression, and cardiovascular diseases. For surgical patients, poor sleep can exacerbate psychological stress, leading to anxiety and insomnia (24–27), which severely harm physical and mental health. Therefore, it is necessary to further investigate the preoperative sleep quality of surgical patients. Patients with rotator cuff injuries often experience shoulder pain and limited mobility. Clinical treatment primarily focuses on alleviating pain and improving shoulder function. However, sleep disturbances in RCT patients have received little attention. Sleep disorders are common among RCT patients. This study, based on patients undergoing ARCR, explores whether preoperative sleep quality affects rapid recovery after ARCR.

High sleep quality helps alleviate postoperative pain after ARCR and reduces the risk of postoperative anxiety and depression. The findings of this study reveal that on postoperative day 1, day 3, and 1 week, the LSQG had significantly higher VAS scores, SAS scores, and SDS scores compared to the HSQG. Additionally, the LSQG exhibited more pronounced postoperative pain, depression, and anxiety. This intergroup difference, influenced by sleep quality, suggests a close relationship between postoperative pain, depression, anxiety, and sleep quality. Previous studies using the PSQI to assess sleep quality in ARCR patients found that patients with higher PSQI scores often experienced poor sleep quality, nighttime insomnia, pain, and anxiety postoperatively (28–30). This indicates that ARCR patients with poor sleep quality are more likely to experience postoperative pain, depression, and anxiety, highlighting the potential link between sleep quality and postoperative pain and anxiety, which aligns with the findings of this study. Further research has shown that patients with higher PSQI scores exhibit more significant postoperative pain and anxiety. However, over time, these symptoms improve, with VAS scores and SAS scores showing continuous improvement and the intergroup gap gradually narrowing (31–33). Consistent with these findings, this study observed significant differences in VAS scores, SAS scores, and SDS scores between the two groups within the first postoperative week. From 1 month to 6 months postoperatively, the intergroup differences gradually diminished, and the time × group interaction effects were statistically significant. This suggests that preoperative high sleep quality helps alleviate postoperative pain and reduces the risk of postoperative anxiety and depression. Using wearable sleep monitoring technology, Ansok et al. (34) prospectively monitored 18 patients with complete rotator cuff tears (RCT). Sleep monitoring data revealed that patients with poor sleep quality experienced prolonged sleep latency, reduced total sleep time, lighter sleep stages, and increased sensitivity to nighttime pain. Similarly, Tanik et al. (35) found that some RCT patients with higher local pressure experienced poor sleep quality, which was more likely to cause local pain and discomfort. These findings underscore the close association between preoperative sleep quality and pain, indicating that improving sleep quality can reduce patients’ sensitivity to postoperative pain. Therefore, improving preoperative sleep quality can help reduce postoperative pain, mitigate the risk of postoperative anxiety and depression, and promote faster recovery in patients.

High sleep quality can reduce postoperative pain after ARCR, promote functional exercise of the affected limb, and facilitate rapid recovery after RCT. Studies have found that poor sleep quality negatively impacts the physical and mental health of RCT patients, increasing their sensitivity to pain (12, 36), which in turn affects limb function and postoperative recovery. The results of this study show that the HSQG experienced significantly less postoperative pain compared to the LSQG. At 1 week, 1 month, and 3 months postoperatively, the ASES scores and Constant-Murley scores for shoulder function were significantly better in the HSQG than in the LSQG. This indicates that preoperative sleep quality is closely related to postoperative pain and shoulder function. Improving sleep quality can reduce patients’ sensitivity to postoperative pain, thereby promoting postoperative functional exercise and achieving rapid recovery. Longo et al. (37) assessed shoulder function in RCT patients by evaluating changes in PSQI scores and found that preoperative and postoperative PSQI scores were closely correlated with ASES scores and Constant-Murley scores. Higher PSQI scores were associated with poorer postoperative shoulder function, while lower PSQI scores were associated with better postoperative shoulder function. This suggests that better sleep quality can alleviate postoperative pain, reduce patients’ sensitivity to pain, and facilitate postoperative functional exercise, leading to rapid recovery. These findings are consistent with the results of this study, further supporting the notion that high sleep quality contributes to rapid recovery after ARCR. Danilkowicz et al. (38) found a high prevalence of sleep dysfunction in RCT patients, which is associated with anxiety, pain, depression, and fatigue. These factors increase postoperative psychological stress, heighten pain sensitivity, and reduce the range of motion in the affected limb, ultimately impairing limb function. This indicates that RCT patients with poor sleep quality are more sensitive to pain. Improving preoperative sleep quality can alleviate postoperative pain. Therefore, enhancing sleep quality can reduce sensitivity to pain after ARCR, promote functional exercise of the affected limb, and achieve rapid postoperative recovery.

Good sleep quality before surgery can enhance patients’ satisfaction during hospitalization, reduce the risks of postoperative joint adhesions and re-tears, and facilitate rapid postoperative recovery. Good sleep quality is closely related to patients’ satisfaction during hospitalization. RCT patients often experience poor sleep quality or even insufficient sleep due to pain, and sleep quality directly affects the mental health status of patients during hospitalization (39). Therefore, the sleep quality of patients before surgery is particularly important. Studies have found that patients who maintain adequate and high-quality sleep before surgery usually feel less anxiety and discomfort during hospitalization, and thus achieve a better psychological state (40, 41). This not only benefits doctor-patient communication but also encourages patients to cooperate actively with treatment, thereby improving the overall experience and satisfaction. Therefore, it is more conducive to patients’ postoperative functional exercise and further reduces the risk of postoperative joint adhesions. The satisfaction survey and postoperative follow-up results in this study show that the proportion of satisfied patients with HSQG is higher, and the occurrence of joint adhesions is also less. This effectively avoids postoperative anxiety and depression, enabling patients to have a better hospital experience. Improving the sleep quality before surgery can reduce the incidence of ARCR complications (42). Previous studies have shown that there is a complex bidirectional relationship between sleep and the immune system. Adequate and effective sleep can enhance immune function and the regenerative capacity of soft tissues (43, 44). During sleep, the body secretes growth factors that promote tissue repair and regeneration (45, 46). Therefore, high-quality sleep can strengthen the immune system, promote the repair and regeneration of postoperative tissues, and effectively reduce the risk of re-tear after surgery. Consistent with previous studies, this study found that the incidence of HSQG rotator cuff re-tear is lower. This further indicates that high-quality sleep before surgery can reduce the occurrence of ARCR complications, effectively promote rapid postoperative recovery, improve the overall surgical outcome, and further enhance patient satisfaction.

### Strengths and limitations of the study

The advantages of this study include the adoption of a prospective cohort study design, a complete follow-up of 256 consecutive patients, an adequate sample size, and the minimization of recall and selection biases. Additionally, in addition to traditional pain and functional scores, psychological scales (SAS/SDS), hospital satisfaction, and complications were also included to comprehensively assess the impact of preoperative sleep quality on the rapid postoperative recovery of patients. However, this study also has some limitations. Firstly, the patient-reported results (VAS, SAS, SDS) are essentially still subjective; although cross-validation by two trained professional evaluators was conducted, residual biases cannot be completely ruled out. Secondly, we excluded patients with hypertension, diabetes, or other major comorbidities to limit biological heterogeneity; therefore, these findings may not be generalized to a broader elderly population with these common diseases. Future research should include patients with a typical comorbidity spectrum for real-world validation.

## Conclusion

In conclusion, high-quality preoperative sleep is beneficial for the postoperative recovery of patients undergoing ARCR. Compared with patients with poor sleep quality, those who had high-quality sleep before surgery experienced less early postoperative pain and psychological distress, earlier functional recovery, higher hospital satisfaction, and fewer complications. These advantages were most pronounced within 3 months after surgery and gradually became consistent thereafter. Therefore, preoperative sleep screening and optimization should be incorporated into the perioperative care of rotator cuff tears to facilitate rapid postoperative recovery for ARCR patients and improve the overall outcome.

## Data Availability

The raw data supporting the conclusions of this article will be made available by the authors, without undue reservation.
